# Comparative cytogenetics of Auchenorrhyncha (Hemiptera, Homoptera): a review

**DOI:** 10.3897/zookeys.538.6724

**Published:** 2015-11-19

**Authors:** Valentina Kuznetsova, Dora Aguin-Pombo

**Affiliations:** 1 Department of Karyosystematics, Zoological Institute of Russian Academy of Sciences, Universitetskaya nab. 1, 199034 St. Petersburg, Russia; 2 Saint Petersburg Scientific Center, Universitetskaya nab. 5, 199034, St. Petersburg, Russia; 3 University of Madeira, 9000-390 Funchal, Madeira Il., Centro de Investigação em Biodiversidade e Recursos Genéticos (CIBIO), Vairão, Portugal

**Keywords:** Chromosome structure, chromosome numbers, sex chromosome systems, B-chromosomes, polyploidy, polymorphism, meiosis, chromosome evolution, Cicadoidea, Cercopoidea, Membracoidea, Myerslopioidea, Fulgoroidea, Cicadomorpha, Fulgoromorpha

## Abstract

A comprehensive review of cytogenetic features is provided for the large hemipteran suborder Auchenorrhyncha, which currently contains approximately 42,000 valid species. This review is based on the analysis of 819 species, 483 genera, and 31 families representing all presently recognized Auchenorrhyncha superfamilies, e.i. Cicadoidea (cicadas), Cercopoidea (spittle bugs), Membracoidea (leafhoppers and treehoppers), Myerslopioidea (ground-dwelling leafhoppers), and Fulgoroidea (planthoppers). History and present status of chromosome studies are described, as well as the structure of chromosomes, chromosome counts, trends and mechanisms of evolution of karyotypes and sex determining systems, their variation at different taxonomic levels and most characteristic (modal) states, occurrence of parthenogenesis, polyploidy, B-chromosomes and chromosome rearrangements, and methods used for cytogenetic analysis of Auchenorrhyncha.

## Introduction

The hemipteran (homopteran) suborder Auchenorrhyncha is divided into two major lineages: the infraorder Cicadomorpha with superfamilies Cicadoidea (cicadas), Cercopoidea (spittle bugs), Membracoidea (leafhoppers and treehoppers), and Myerslopioidea (ground-dwelling leafhoppers), and the infraorder Fulgoromorpha with the single superfamily Fulgoroidea (planthoppers) (Szwedo et al. 2004, Aguin-Pombo and Bourgoin 2012). More than 42,000 valid species of Auchenorrhyncha have been reported worldwide (Deitz 2008), which, depending on the classification followed, can be grouped roughly into 30 to 40 families.

Olli Halkka (Halkka 1959), one of the earliest and most well-known researchers of chromosomes in Auchenorrhyncha, concluded that they “are a group well suited for comparative karyological work. Technically, this group presents no special difficulties. The numbers of the chromosomes are relatively low and the chromosomes themselves are fairly large”. The first cytogenetic studies on Auchenorrhyncha provided data for the cicada species *Diceroprocta tibicen* Linnaeus (Cicadidae) (Wilcox 1895) and the spittlebug species *Lepyronia quadrangularis* Say (Aphrophoridae) (Stevens 1906). Shortly afterwards Boring (1907) initiated research on the comparative karyology of Auchenorrhyncha with a study of 22 species belonging to five families. Documented lists of Auchenorrhyncha chromosome numbers have been published by several authors. Those of Halkka (1959) and Kirillova (1986, 1987) cover the complete suborder Auchenorrhyncha. The first author discussed different aspects of auchenorrhynchan cytogenetics, while the second reported only chromosome numbers and sex determining systems. Later, lists for particular fulgoromorphan families were published by Kuznetsova et al. (1998) for Cixiidae, Meenoplidae, Derbidae, Achilidae, Nogodinidae, Tropiduchidae, and Flatidae; by Maryańska-Nadachowska et al. (2006) for Issidae, Caliscelidae, and Acanaloniidae; by Kuznetsova et al. (2009a) for Dictyopharidae and Fulgoridae; and then, Tian et al. (2004) added data for 19 species of the families Cixiidae, Delphacidae, Fulgoridae, Ricaniidae, Issidae, Flatidae, and Achilidae, while Kuznetsova et al. (2010) for 14 species of the issid tribe Issini. In contrast to Fulgoromorpha, the data on cicadomorphan families have never been tabulated after the comprehensive reviews by Halkka (1959) and Kirillova (1986, 1987). Quite recently, chromosome numbers were reported for 91 species of Cicadellidae (Wei 2010, Juan 2011) and 25 species of Membracidae (Tian and Yuan 1997) from China. Several additional species were also karyotyped within the families Cicadidae, Cercopidae, Aphrophoridae, Cicadellidae, and Myerslopiidae (Marin-Morales et al. 2002, Perepelov et al. 2002, Kuznetsova et al. 2003, 2013, 2015a, Aguin-Pombo et al. 2006, 2007, Maryańska-Nadachowska et al. 2008, 2012, Castanhole et al. 2010, de Bigliardo et al. 2011, Golub et al. 2014).

**Figure 1.**
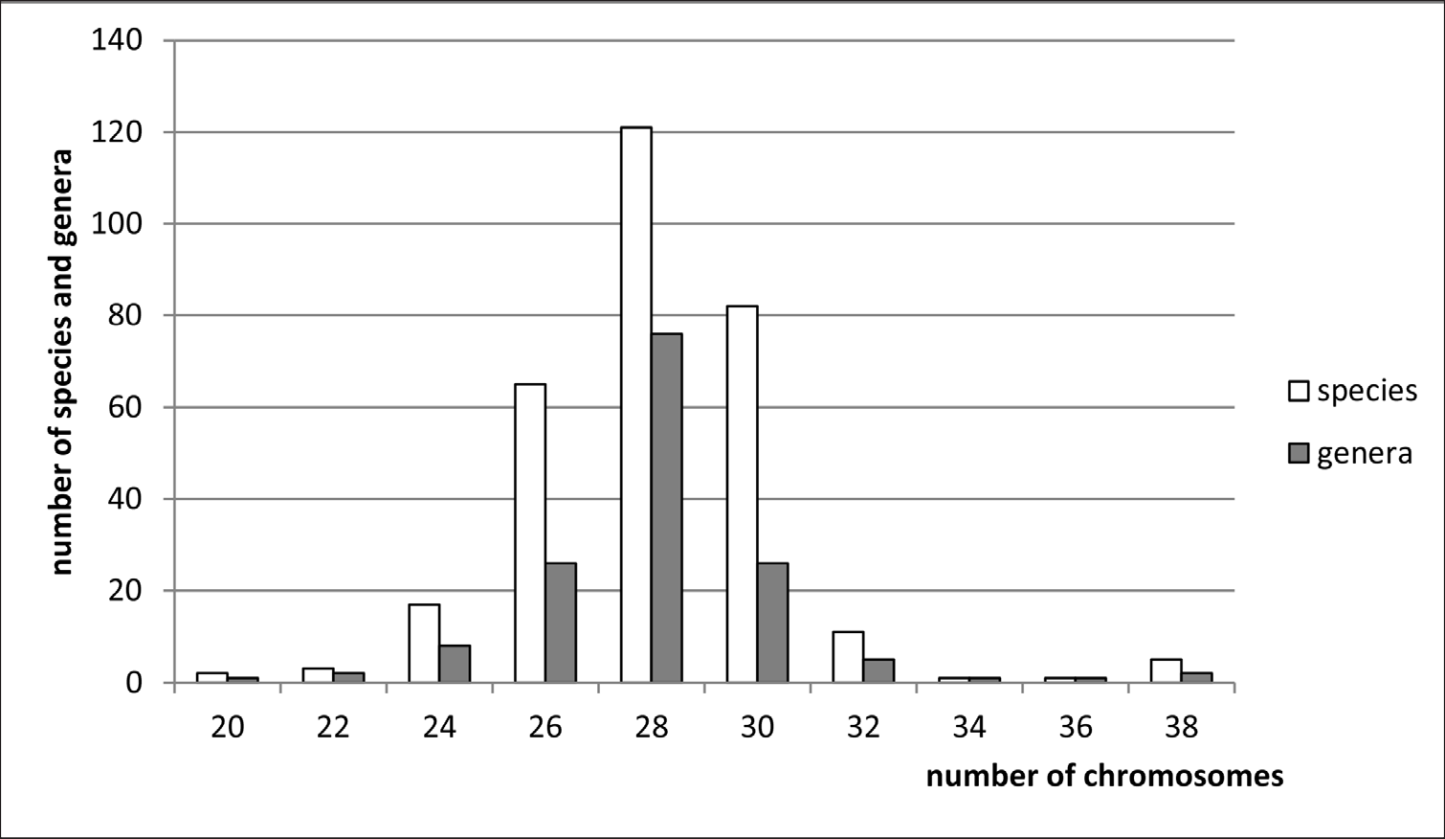
Histogram showing the distribution of female diploid chromosome numbers in Fulgoroidea at species and generic levels, based on analysis of 308 species and 148 genera of the families Tettigometridae, Delphacidae, Cixiidae, Kinnaridae, Meenoplidae, Derbidae, Achilidae, Achilixiidae, Dictyopharidae, Fulgoridae, Issidae, Caliscelidae, Acanaloniidae, Nogodinidae, Ricaniidae, Flatidae, Hypochthonellidae, Lophopidae, Eurybrachyidae, and Gengidae.

**Figure 2.**
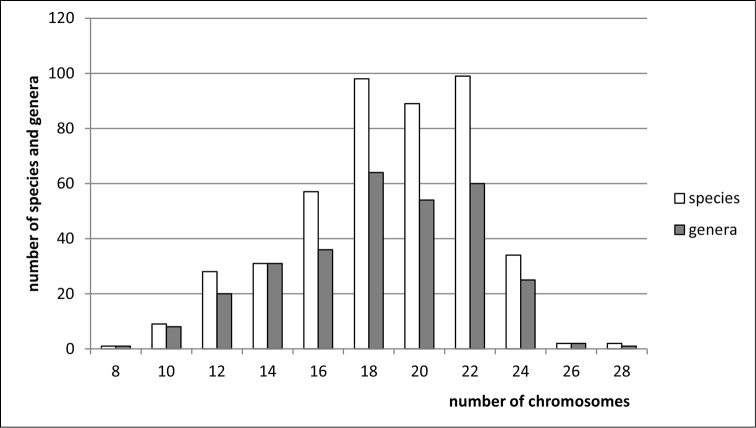
Histogram showing the distribution of female diploid chromosome numbers in Membracoidea at species and generic levels, based on analysis of 450 species and 302 genera of the families Cicadellidae, Membracidae, Ulopidae, Ledridae, and Aetalionidae.

**Figure 3.**
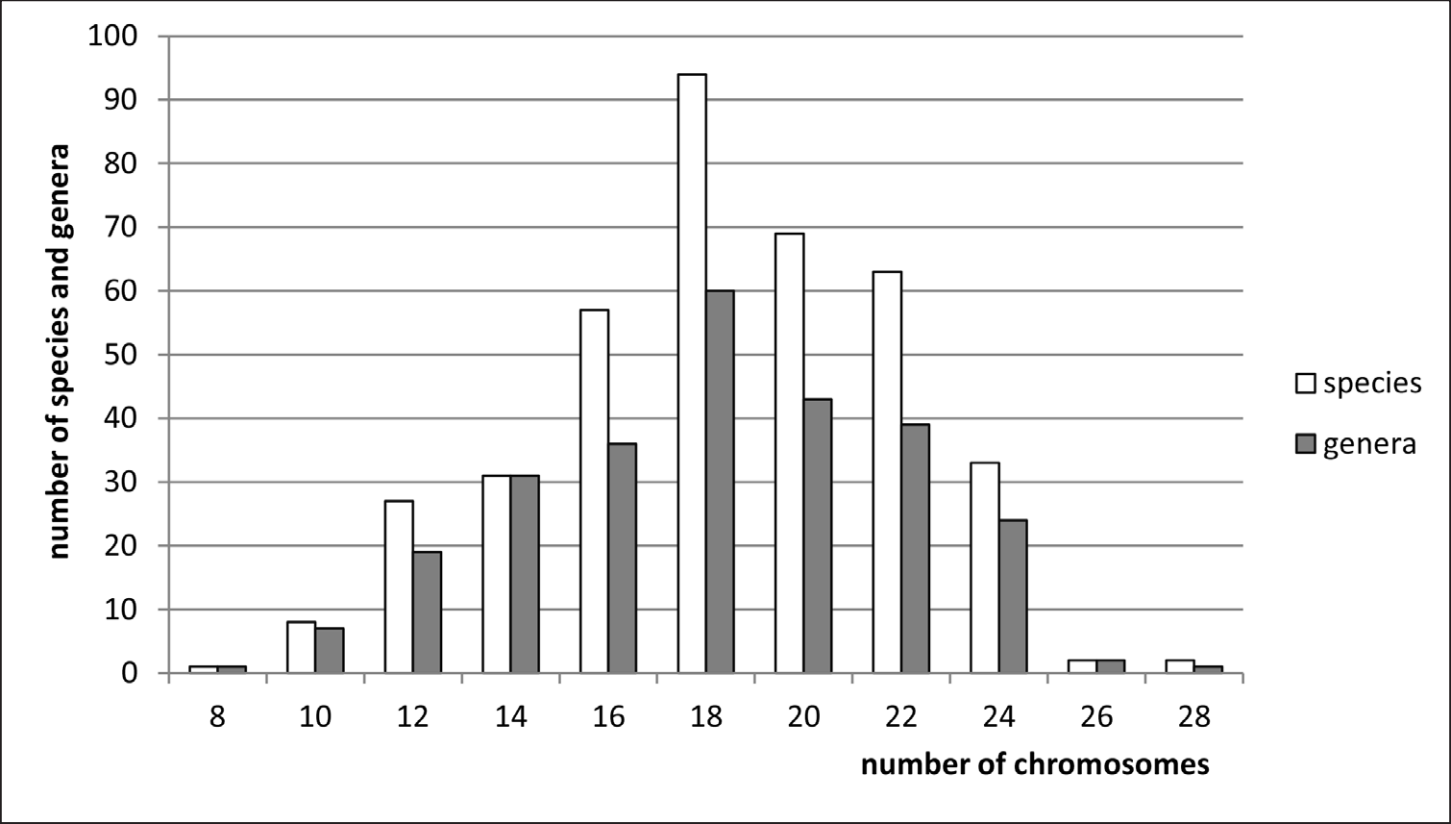
Histogram showing the distribution of female diploid chromosome numbers in Cicadellidae at species and generic levels, based on analysis of 387 species and 263 genera.

**Figure 4.**
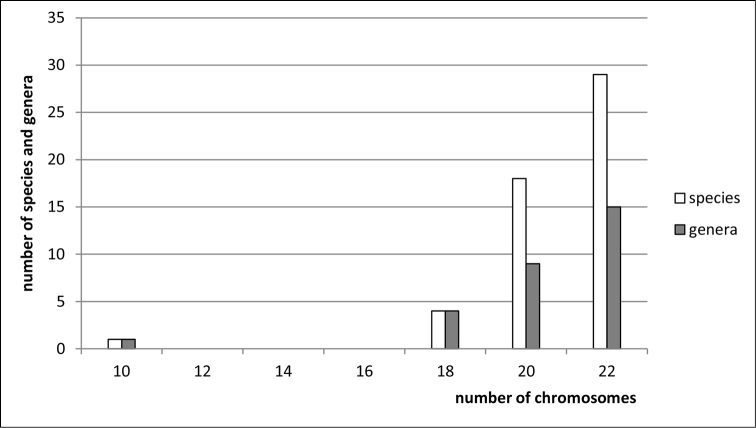
Histogram showing the distribution of female diploid chromosome numbers in Membracidae at species and generic levels, based on analysis of 52 species and 29 genera.

**Figure 5.**
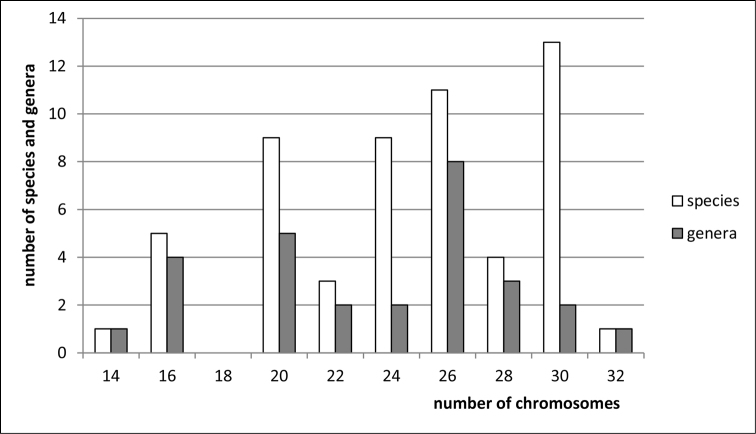
Histogram showing the distribution of female diploid chromosome numbers in Cercopoidea at species and generic levels, based on analysis of 50 species and 23 genera of the families Cercopidae, Aphrophoridae, Machaerotidae, and Clastopteridae.

**Figure 6.**
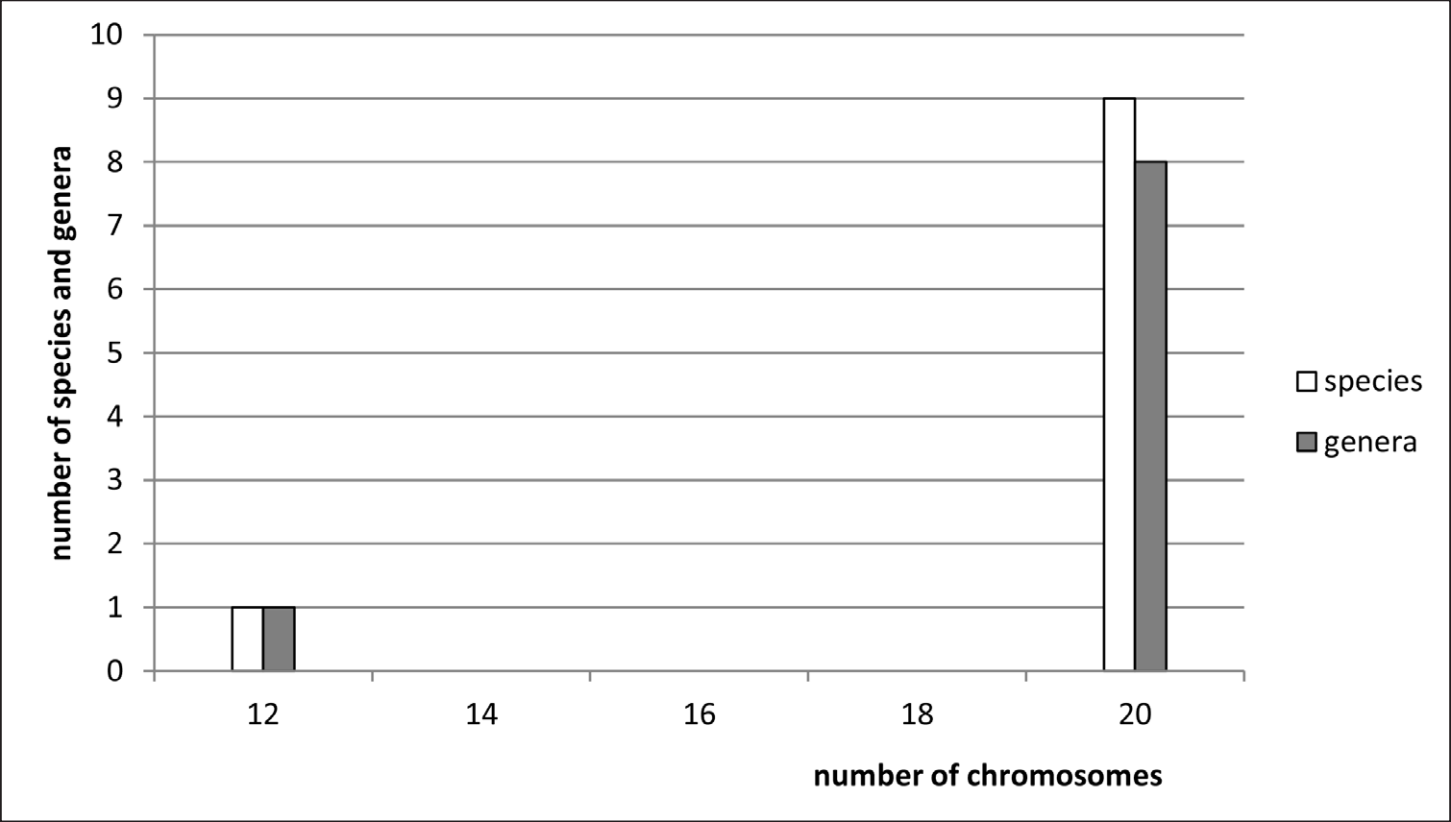
Histogram showing the distribution of female diploid chromosome numbers in Cicadoidea at species and generic levels, based on analysis of 10 species and 9 genera of the family Cicadidae.

**Figure 7.**
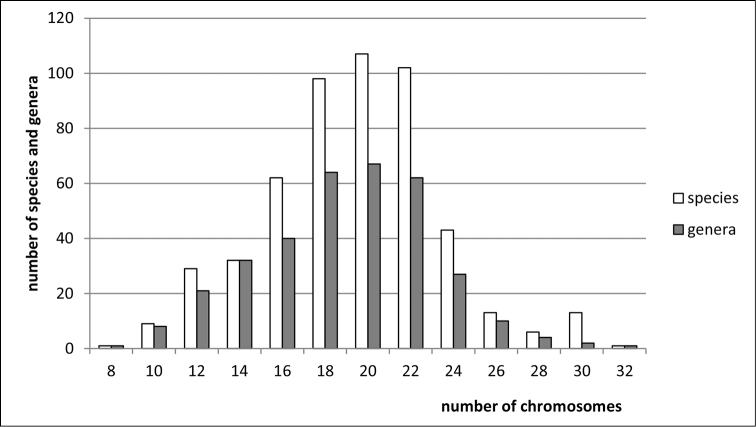
Histogram showing the distribution of female diploid chromosome numbers in Cicadomorpha at species and generic levels, based on analysis of 511 species and 335 genera of the families Cicadellidae, Membracidae, Ulopidae, Ledridae, Aetalionidae, Cercopidae, Aphrophoridae, Machaerotidae, Clastopteridae, Cicadidae, and Myerslopiidae.

**Figure 8.**
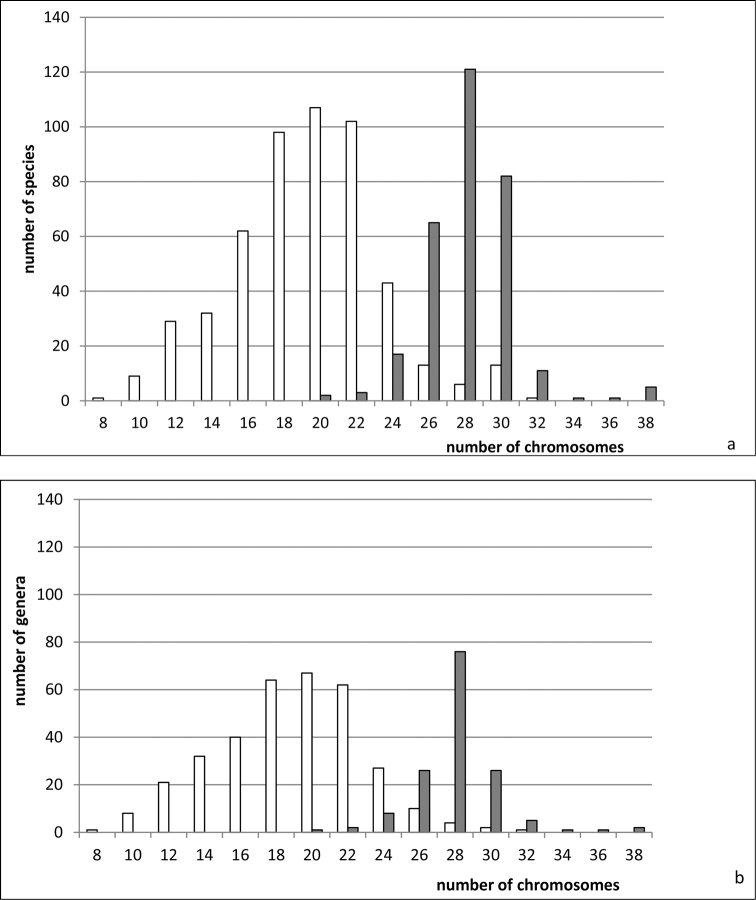
Histograms showing the distribution of female diploid chromosome numbers in Cicadomorpha (**a, b** white columns) and Fulgoromorpha (**a, b** black columns) at species (**a**) and generic (**b**) levels, based on analysis of 819 species and 483 genera.

At the present time, approximately 819 auchenorrhynchan species (nearly 2% of the total number of species described) are known from a cytogenetic viewpoint (V. Kuznetsova, unpublished checklist). These species represent 483 genera and 31 families from all the superfamilies of Auchenorrhyncha. Of these taxa, 511 species, 335 genera and 11 families belong to Cicadomorpha, while 308 species, 148 genera, and 20 families belong to Fulgoromorpha (Figs 1–8). The available data were chiefly obtained using conventional cytogenetic techniques and concerned, almost entirely, chromosome numbers, sex determining systems, and, in outline, the behaviour of chromosomes during meiosis. A few recent studies have used modern cytogenetic techniques to identify the individual chromosomes in karyotypes and specific regions in chromosomes of auchenorrhynchan species (Kuznetsova et al. 2003, 2009b, 2010, 2015a, Maryańska-Nadachowska et al. 2008, 2012, 2013, Golub et al. 2014). The application of new techniques, primarily fluorescence *in situ* hybridization (FISH), opened a promising area of research, which yields more detailed karyotype information (Maryańska-Nadachowska et al. 2013, Golub et al. 2014, Kuznetsova et al. 2015a).

Since the Halkka’s (1959) excellent review, the comparative cytogenetics of Auchenorrhyncha has never been rigorously addressed. The only exception is the two-part paper of Emeljanov and Kirillova (1990, 1992), which presents a comprehensive analysis of chromosome numbers and their variation at different taxonomic levels within every auchenorrhynchan family explored at that time. Thus, nearly fifty five years after Halkka’s and twenty five years after Emeljanov and Kirillova’s publications, we discuss here different aspects of cytogenetics of Auchenorrhyncha and summarize progress and problems in the field.

## Chromosome structure

The overwhelming majority of eukaryotic organisms have monocentric chromosomes. These chromosomes possess the localized centromere, a region where two chromatids join and where spindle fibers attach during mitosis and meiosis. Like all Hemiptera, Auchenorrhyncha have holokinetic (holocentric) chromosomes. In contrast to monocentric chromosomes, holokinetic chromosomes have no localized centromere. The latter is considered to be diffuse and is formed by a large kinetochore plate (a circular plaque structure on the centromere by which the chromosomes are attached to spindle polar fibers) extending along all or most of the length of the holokinetic chromosome (Schrader 1947, Wolf 1996). Holokinetic chromosomes are sometimes designated as holocentric despite the fact that they lack a proper centromere. These chromosomes occur in certain scattered groups of plants and animals, being particularly widespread in insects, including Odonata (Palaeoptera), Dermaptera (Polyneoptera), Psocoptera, Phthiraptera, Hemiptera (Paraneoptera), Lepidoptera, Trichoptera (Oligoneoptera) (White 1973), and the enigmatic Zoraptera (Kuznetsova et al. 2002). Thus, holokinetic chromosomes occur in every major phylogenetic lineage (cohort) of Pterygota suggesting that they are likely to have evolved at least four times independently in insect evolution.

In theory, the large kinetochore plate facilitates rapid karyotype evolution via occasional fusion/fission events. Firstly, fusion of holokinetic chromosomes would not create the problems characteristic of a dicentric chromosome in monocentric organisms (i.e. displaying chromosomes with localized centromeres). Secondly, fission of a holokinetic chromosome should create chromosome fragments that exhibit a part of the kinetochore plate and can attach themselves to the spindle fibers at cell divisions. As a result, chromosome fragments that would be acentric (lacking a centromere) and hence lost in organisms with monocentric chromosomes may be inherited in holokinetic organisms. The gametes harboring chromosome fragments are consequently expected to be viable (Hipp et al. 2010). Fusion/fission rearrangements are therefore conventionally accepted as the commonest mechanisms of chromosome evolution in holokinetic groups. This assumption seems to receive support from the fact that the greatest range of within-genus variation in chromosome number related to the fusion/fission rearrangements is described in organisms with holokinetic chromosomes (reviewed in Kuznetsova et al. 2011). The evidence for the unique potential of holokinetic chromosomes’ fissions is provided by the blue butterfly *Polyommatus atlanticus* Elwes (Lycaenidae), 2n = ca 448-452, holding the record of the highest number of chromosomes in the non-polyploid eukaryotic organisms (Lukhtanov 2015).

Although variations in chromosome number of related species are probably due to both fissions and fusions, fusions are suggested to be more common in holokinetic groups (White 1973) including Auchenorrhyncha (Halkka 1959, 1964). The point is that a chromosome, whether holokinetic or monocentric, has to display two functional telomeres in order to survive a mitotic cycle. A chromosome resulting from a fusion event will always display two functional telomeres originated from the two ancestral chromosomes, whereas a chromosome from a fission event will have to be able to develop a functional telomere *de novo* (White 1973, Nokkala et al. 2007).

### Chromosome numbers and possible trends of their evolution

**Variation in chromosome number.** The currently known diploid chromosome numbers in Auchenorrhyncha range between 8 and 38 (here and elsewhere chromosome numbers are provided for females), being the lowest in Cicadomorpha (Cicadellidae) and the highest in Fulgoromorpha (Delphacidae and Dictyopharidae). The infraorders differ in the limits of variation in chromosome number and in the modal numbers (sometimes referred to as the type numbers or basic numbers). Within each infraorder, many taxa have more than one modal number and these are characteristically lower in Cicadomorpha than in Fulgoromorpha. In Cicadomorpha, chromosome numbers vary from 2n = 8 (*Orosius* sp. from Cicadellidae) to 2n = 32 (*Peuceptyelus coriaceus* Fallén from Aphrophoridae). The numbers in most cicadomorphan species lie between 16 and 22, with rare exceptions above and below these limits. In Fulgoromorpha, chromosome numbers vary from 2n = 20 (*Pentastiridius hodgarti* Distant from Cixiidae) to 2n = 38 (*Scolops* spp. from Dictyopharidae and *Paraliburnia clypealis* Sahlberg from Delphacidae) with strongly marked modes at 28 (prevailing), 30 (second) and 26. The variation in chromosome number in various groups of Auchenorrhyncha is shown in Figs 1–8.

Despite the fact that all Auchenorrhyncha possess holokinetic chromosomes, many higher taxa of the suborder show stable or only slightly variable karyotypes. Quite often the chromosome number is constant within the same genus. Within Cicadellidae, the genera *Eurymela* Le Peletier & Serville, *Eurymeloides* Ashmead, and *Cicadula* Zetterstedt are examples. In the first, all three studied species, *Eurymela distincta* Signoret, *Eurymela erythrocnemis* Burmeister, and *Eurymela fenestrata* Peletier & Serville, share 2n = 22; in the second, all four studied species, *Eurymela bicincta* Erichson, *Eurymela perpusilla* Walker, *Eurymela pulchra* Signoret, and *Eurymela punctata* Signoret, possess likewise 2n = 22; in the third, four studied species, *Cicadula intermedia* Boheman, *Cicadula quadrinotata* Fabricius, *Cicadula persimilis* Edwards, and *Cicadula saturata* Edwards, possess 2n = 16 (de Lello et al. 1982, Kirillova 1987). In the family Aphrophoridae, the large genus *Aphrophora* Germar is characterized by 2n = 30, whereas in the Membracidae, the majority of species in the genera *Gargara* Amyot & Serville and *Leptocentrus* Stål shows 2n = 20 and 2n = 22, respectively (Kirillova 1987). The most impressive examples of chromosome stability come from groups which have been extensively studied. Thus, nearly all species and genera of the subfamily Eurymelinae (Cicadellidae) have 2n = 22 (Whitten 1965), while those (33 species) of the tribe Issini (Issidae) – 2n = 28 (Maryańska-Nadachowska et al. 2006, Kuznetsova et al. 2010). Similarly, within the family Dictyopharidae, almost all so far studied representatives of the tribe Dictyopharini (9 species) have 2n = 30; those of the tribe Ranissini (8 species) 2n = 28, while those of the tribe Almanini (16 species) – 2n = 26 (Kuznetsova 1986, Kuznetsova et al. 2009a). The conservative numbers suggest no evidence that fusions/fissions have played a role in speciation and evolution of these groups.

By contrast, there are some groups in which a wide variety of chromosome numbers occurs suggesting that both fusions and fissions have established themselves during their evolution. In Cicadellidae, within the genus *Eurhadina* Haupt, the 19 studied species vary broadly in chromosome number: 2n = 12, 14, 16, 18, and 20 (Halkka 1957, Juan 2011). The genus *Empoasca* Walsh is another group, which seems to show a striking range in chromosome number. In this genus, the twelve species examined so far display 2n = 16 (4 species), 18 (2 species), 20 (4 species), and 22 (2 species) (Kirillova 1988, Aguin-Pombo et al. 2006, Juan 2011). This cosmopolitan genus with more than 1,000 described species is by far the most speciose genus in Cicadellidae. *Empoasca* is recognized as a genus requiring comprehensive revision (Southern and Dietrich 2010), and a cytogenetic approach might be useful to clarify the species-level systematics of this group. Likewise, the eight species recognized in the remarkably polymorphic spittlebug genus *Philaenus* Stål (Aphrophoridae) display three different chromosome numbers in males: 20, 23, and 24 (Maryańska-Nadachowska et al. 2012).

**The modal and ancestral chromosome numbers.** Emeljanov and Kirillova (1992) argued that the ancestral chromosome numbers were as follows: 2n = 30 in Fulgoroidea, 2n = 26-28 in Cercopoidea, 2n = 20 in Cicadoidea, and 2n = 22 in Membracoidea. Although 2n = 22, discovered later in the most primitive membracoid family Aetalionidae (Kuznetsova and Kirillova 1993), seemed to confirm the ancestrality of this number in Membracoidea, a more definite solution of this problem will be possible only after a more thorough investigation of every superfamily. For example, the currently available data on Fulgoroidea (308 species and 148 genera) are greatly skewed owing to the focus on the families Dictyopharidae and Delphacidae and on the tribe Issini (Issidae), in which altogether over 160 species in 122 genera have been karyotyped. In Cercopoidea, chromosome numbers are known for 50 species (23 genera), half of which belong to the family Aphrophoridae, while in Cicadoidea only ten species (nine genera) in the Cicadidae have been studied. Within Membracoidea, 450 species in 302 genera have been karyotyped, of which at least 65% of species and 53% of genera belong to the subfamilies Typhlocybinae and Deltocephalinae (Cicadellidae). It should be added here that *Mapuchea chilensis* Nielson, the recently studied first representative of the cicadomorphan superfamily Myerslopoidea (Myerslopiidae), was found to exhibit 2n = 18(16 + XY) (Golub et al. 2014). This chromosome number fits well into the range of most characteristic numbers in Cicadomorpha as a whole (16-22) being close to the numbers accepted by Emeljanov and Kirillova (1992) as putative ancestral ones for Cicadoidea (2n = 20) and Membracoidea (2n = 22) but not for Cercopoidea (2n = 26-28).

Opinions on the ancestral chromosome number in Auchenorrhyncha as a whole differ considerably (Halkka 1959, Kuznetsova et al. 1998, Emeljanov and Kirillova 1992). The solution of the problem very much depends on phylogeny accepted and method of ancestral number inference adopted. One approach to inferring the ancestral karyotype is mapping chromosome numbers typical for particular superfamilies on phylogenetic trees. Some researchers treat Fulgoroidea (Fulgoromorpha) as the most basal branch within Auchenorrhyncha (e.g. Shcherbakov 1984). This idea recently supported by molecular data (Cryan and Urban 2012) is tempting to speculate that the typical number for Fulgoromorpha, 2n = 30 (Halkka 1959) or 2n = 28 (Kuznetsova et al. 1998), is the ancestral state in Auchenorrhyncha. However, based on a large number of morphological characters, Emeljanov (1987) hypothesized that the common ancestor of the four (now five including Myerslopioidea) recent superfamilies differentiated first into cercopoid-cicadoid and fulgoroid-cicadelloid branches, thus becoming the clade Cercopoidea + Cicadoidea, the sister group of the clade Fulgoroidea + Membracoidea. Comparison of the putative ancestral numbers of the superfamilies (see above) with the Emeljanov’s phylogenetic scheme of Cercopoidea + Cicadoidea suggests that the characteristic karyotype of Cercopoidea, 2n = 28 or 26, would be the most likely ancestral chromosome number in Auchenorrhyncha as a whole (Emeljanov and Kirillova 1992). Another approach to inferring the ancestral karyotype is a comparison of typical chromosome numbers between the target group and outgroups. Emeljanov and Kirillova (1992) used this approach and compared chromosome numbers in Auchenorrhyncha with those in other hemipteran lineages – Aphidomorpha, Coccomorpha, Psyllomorpha, Aleyrodomorpha, and Heteroptera. This led to the conclusion that the common ancestor of all Auchenorrhyncha had a diploid set of 20-22 chromosomes.

Since Emeljanov and Kirillova’s (1992) publication, a large body of new cytogenetic data on hemipterans has been obtained. There is a good reason to reconsider the problem based on the data available at present time. In Coccomorpha, diploid numbers vary from 4 to 192, with comparatively low numbers in “archaeococcoids” comprising the most basal families of scale insects – Ortheziidae (2n = 14, 16, 18), Margarodidae (modal number of 2n = 4), and Phenacoleachiidae (2n = 8 in the only studied species *Phenacoleachia zealandica* Cockerell) (Gavrilov 2007). In Aphidomorpha, chromosome numbers vary between 4 and 72, the ancestral number most likely being between 8 and 20 (Blackman 1980) though, in our opinion, more likely between 8 and 22. In the most primitive families, 2n = 22 (Adelgidae), 2n = 8 and 12 (Phylloxeridae) and 2n = 20 (Eriosomatidae = Pemphigidae) seem to be most characteristic (Kuznetsova and Shaposhnikov 1973, Blackman 1980, Gavrilov-Zimin et al. 2015), but the sampling is still inadequate, at least for Adelgidae and Phylloxeridae (Gavrilov-Zimin et al. 2015). Although it is not necessary for a modal number to be ancestral in a group, it seems reasonable to assume that in Psyllomorpha the karyotype of 2n = 26, which has been conserved in 72% of the species and in 50% of the genera studied, is their ancestral trait (Maryańska-Nadachowska 2002). In Aleyrodomorpha, chromosome numbers are known for only four species (see Blackman and Cahill 1998): *Trialeurodes vaporariorum* Westwood (2n = 22), *Aleurotulus nephrolepidis* Quaintance (2n = 26 and/or 28), *Aleurodes proletella* Linnaeus (2n = 26), and *Bemisia tabaci* Gennadius (2n = 20). In one of the most primitive true bug infraorder, the Dipsocoromorpha, chromosome numbers have been recorded for males of three representatives of the family Dipsocoridae: 2n = 20 + X(0) in *Cryptostemma rufescens* Sahlberg, 2n = 20 + XY_1_Y_2_ in *Cryptostemma pussillimum* Sahlberg, 2n = 20 + XY in *Cryptostemma hickmani* Hill, 2n = 20? + XY in *Cryptostemma castaneovitreus* Linnavuori, and for males of two species of the family Schizopteridae: 2n = 32 + X(0) in *Pateena elimata* Hill and *Rectilamina australis* Hill (Grozeva and Nokkala 1996). For the two studied Coleorrhyncha species, *Xenophyes cascus* Bergroth and *Peloridium pomponorum* Shcherbakov, male karyotypes of 2n = 26 + X(0) and 2n = 30 + X(0) respectively were recorded (Grozeva et al. 2014, Kuznetsova et al. 2015b).

In Psocomorpha, a sister group to the rest of Paraneoptera, the modal karyotype of 2n = 18 is considered as the ancestral one, although there appears to be considerable variation in chromosome number within more primitive suborder Trogiomorpha: 2n = 18 and 22 in Trogiidae, 2n = 20 in Psoquillidae, and 2n = 30 in Psyllipsocidae (Golub and Nokkala 2009). Thus, the available data on the higher hemipteran groups appear insufficient and too heterogeneous to reconstruct the chromosome number ancestral for Auchenorrhyncha.

## Sex determining systems

Genetic sex determination predominates in higher animals, including insects, and is often accompanied by the presence of a heteromorphic chromosome pair in one sex (White 1973). The Auchenorrhyncha, in common with most other insects, display male heterogamety. The XX/X(0) sex determination (where 0 denotes the absence of the Y chromosome) is of common occurrence and seems to be an ancestral trait in this group (Halkka 1959, Emeljanov and Kirillova 1990, 1992) and in Hemiptera as a whole (Blackman 1995). Thus, in females two Xs are present, while in males only one X is present. Despite evolutionary stability, in some cases the X(0) system has been replaced by an XY system in species within the same genus that is otherwise exclusively X(0). When such cases occur in Auchenorrhyncha, it is often clear that the Y is a neo-Y (Blackman 1995). Examples of this are found in the genera *Oncopsis* Burmeister from Cicadellidae (John and Claridge 1974) and *Philaenus* from Aphrophoridae (Maryańska-Nadachowska et al. 2012). A highly peculiar situation occurs in the cytologically well-studied tribe Almanini (Dictyopharidae), in which all species have a neo-XY system and the only exception is *Almana longipes* Dufour with an X(0) system in males. In contrast to Almanini, species from all other tribes of this family are characterized by an X(0) system (Kuznetsova 1986, Kuznetsova et al. 2009a).

In organisms with XY systems, recombination between X and Y chromosomes is usually suppressed (White 1973) except for the cases when the XY is a neo-system. This type of sex determination usually arises from the ancestral X(0) system as a result of fusion between the original X chromosome and an autosome, the homologue of this autosome becoming a neo-Y chromosome (White 1973, Blackman 1995). Clearly, the derived karyotype should have one pair of autosomes less than the ancestral one. For example, the species of the tribe Almanini (Dictyopharidae) have 2n = 26 + neo-XY, whereas those of the tribe Ranissini 2n = 28 + X(0). In a recently formed neo-XY system, the autosomally derived Y chromosome (a neo-Y) and the autosomal part of the neo-X chromosome are still homologous, and therefore synapse in prophase I of meiosis. At metaphase I, the neo-XY bivalent is usually large and clearly heteromorphic, indicating a recent fusion between the X and an autosome pair.

Once a neo-XY system has arisen, it can undergo a further transformation into a multiple X_1_X_2_Y system as a result of a translocation involving the Y chromosome and another pair of autosomes. This may have occurred in the evolution of the sex chromosome mechanism in *Philaenus italosignus* Drosopoulos & Remane, which has 2n = 20 + neo-X_1_X_2_Y against 2n = 22 + neo XY found in *Philaenus signatus* Melichar, *Philaenus tarifa* Remane & Drosopoulos, and *Philaenus maghresignus* Drosopoulos & Remane (Maryańska-Nadachowska et al. 2012, 2013). Multiple sex chromosomes of the X_1_X_2_Y type, which form chiasmate trivalents in meiosis, have also been described in *Austragalloides* sp. (Cicadellidae), however, in this case the X_1_X_2_Y system represents an example of sex chromosome polymorphism (Whitten 1968).

A different, achiasmate XY system, with a fairly small Y chromosome, is found in the planthoppers *Limois emelianovi* Oshanin and *Limois kikuchii* Kato (Fulgoridae) (Kuznetsova 1986, Tian et al. 2004). The origin of the Y chromosome in these species is not entirely clear, but it seems likely that it has been derived from a mitotically stable B chromosome that has become a standard member of the karyotype, that is, the B chromosome transformed into the Y chromosome during evolution (discussed in section “**Polymorphism for B-chromosomes**”).

### Polyploidy

Polyploidy, that is, multiplication of the chromosome set is well known to play a major role in speciation and evolution of plants, but is a fairly rare phenomenon in sexually reproducing animals (White 1973). Evolutionary polyploidy had occurred in a number of animal species that reproduce parthenogenically. In Auchenorrhyncha, all so far known cases of polyploidy are connected with parthenogenesis, either with gynogenesis, sometimes referred to “pseudogamy” (where the egg is activated by sperm borrowed from conspecific or closely related males, but without fusion of the egg and sperm nuclei), or with true parthenogenesis, more often referred to “thelytoky”. Although it is not necessary for all parthenogenetic forms to be polyploids, these are universally triploids in both leafhoppers and planthoppers. Only two planthopper genera, *Muellerianella* Wagner and *Ribautodelphax* Wagner (Delphacidae), are known to comprise a number of gynogenetic triploid forms (Drosopoulos 1976, 1977, den Bieman and Eggers-Schumacher 1986). In contrast, leafhoppers of the genus *Empoasca* (Aguin-Pombo et al. 2006) and planthoppers of the genus *Delphacodes* Fieber (den Bieman and de Vrijer 1987) comprise triploid forms, which reproduce by true parthenogenesis. The genus *Empoasca* is a good case in point. In this diverse, complex and cosmopolitan genus, the bisexual species are diploid, with hitherto known chromosome numbers of 2n = 16, 18, 20 and 22. In Madeira Island, besides the bisexual species *Empoasca decedens* Paoli (2n = 14 + XX), *Empoasca alsiosa* Ribaut (2n = 16 + XX) and *Empoasca fabalis* DeLong (2n = 20 + XX), three all-female morphotypes (A, B and C) were discovered. In these females, the chromosomal complements are triploid, consisting thus of two female genomes and one male genome: 2n = 3x = 28 + XXX in morphotype A, 24 + XXX in morphotype B, and 21 + XXX in morphotype C. The study revealed that their reproduction follows an apomictic type (Aguin-Pombo et al. 2006).

In apomictic parthenogenesis, meiosis is completely suppressed, and eggs pass through a mitosis-like cell division, i.e. without formation of bivalents and recombination, and genetic heterozygosity is thus preserved. The heterozygosity is expected to be perpetuated from generation to generation, increasing slightly through mutations. It is generally proposed that most polyploid animals are allopolyploids, tending to be of hybrid origin (White 1973, Bullini 1985). It is assumed that such is most likely the case of the triploid forms of *Muellerianella* (Drosopoulos 1976). In contrast, in some groups, triploids seem to have an autopolyploid origin and this is probably true for triploids found in the genus *Ribautodelphax* (den Bieman 1988). The origin of the above-listed *Empoasca* parthenoforms still remains unknown. Some of them seem to be closely related to bisexual species which are yet extant. Several hypotheses, including that of their hybrid origin, were made in Aguin-Pombo et al. (2006) but still much more information is needed to decide between the hypotheses.

### Polymorphism for B-chromosomes

B-chromosomes (also referred to as supernumerary, additional or accessory) are chromosomes found in addition to chromosomes of the standard complement (A chromosomes) and occur in approximately 15% of living species (Beukeboom 1994). Little consensus has been achieved in understanding their origin, role, transmission, inheritance and evolution. B-chromosomes appear only in some individuals of some populations of the same species. Their presence is considered to be beneficial, harmful or neutral, and several authors consider B-chromosomes as parasitic and selfish (reviewed in Camacho 2004).

Small chromosomes additional to the standard complements and interpreted as B-chromosomes have been found in the leafhoppers *Alebra albostriella* Fallen and *Alebra wahlbergi* Boheman (Kuznetsova et al. 2013) and in several species of planthoppers (Halkka 1959, Booij 1982, den Bieman 1988, Kirillova and Kuznetsova 1990). Males of *Alebra albostriella* and *Alebra wahlbergi* were collected from a range of food plants in different localities in Greece. B-chromosomes were found in 3 out of 6 populations of *Alebra albostriella* and in 2 out of 7 populations of *Alebra wahlbergi*. A single B chromosome or sometimes two B-chromosomes were present in males. As is often the case, B-chromosomes were significantly smaller than chromosomes of the standard complements and negatively heteropycnotic during meiotic prophases and metaphases. No correlation was found between the occurrence and frequencies of B-chromosomes in populations with habitat or altitude (Kuznetsova et al. 2013).

It is suggested that inter-population differences in B chromosome distribution depend on selective factors (Camacho 2004). It is interesting to note in this connection that in the planthopper species *Javesella pellucida* Fabricius, *Criomorphus borealis* Sahlberg, and *Saccharosydne procera* Matsui (Delphacidae), B-chromosomes were present only in populations inhabiting Northeast Siberia and Kamchatka, whereas individuals sampled from different populations in European Russia (e.g. those of *Javesella pellucida*) lacked B-chromosomes (Kirillova and Kuznetsova 1990). The point that should be mentioned is that both these planthopper species and aforesaid *Alebra* Fieber leafhoppers showed no more than 1 or 2 B-chromosomes per individual. One must suggest that this number is tolerable for B chromosome carriers and the natural selection operates to eliminate individuals with more than two B-chromosomes in all these populations.

The commonly accepted view is that B-chromosomes are derived from the standard complement of a species, including the X chromosome (Camacho 2004). On the other hand, the possibility of integrating a B chromosome into the standard chromosome complement has been suggested in a number of studies (reviewed in Nokkala et al. 2000). Recently, it has been claimed that the achiasmate Y chromosome in *Drosophila* Fallén (Diptera) might have evolved from a B chromosome (Carvalho 2002). To explain the formation of the achiasmate Y chromosome in separate species of psyllids (a related group sharing predominant X(0) sex determining system with Auchenorrhyncha), Nokkala et al. (2000, 2003) suggested that the Y chromosome has evolved from a mitotically stable B chromosome that was integrated into an achiasmate segregation system with the X chromosome. Later, this chromosome would become fixed in the karyotype as the Y chromosome. In this connection, it should be noted that at metaphase I of some delphacid species, a B chromosome appeared closely associated with the univalent X chromosome forming a pseudo-bivalent XB (Kirillova and Kuznetsova 1990). The X and B-chromosomes tended to segregate (i.e. pass to opposite poles) at anaphase I suggesting thus an increasing transmission of B-chromosomes to sons while a decreased transmission to daughters. This means that the B-chromosomes are able to spread through the male line, whereas are removed from the female line. In this connection, it is interesting to note that a Y chromosome of unknown origin has been described in several species of the family Fulgoridae (Kuznetsova 1986, Kuznetsova et al. 2010, see also section “**Sex determining systems**”). In these species, the XY chromosome pair was located outside the autosomal bivalents in the place where a univalent X chromosome is usually located in the X(0) auchenorrhynchan species. The XY pair differed distinctly from a neo-XY bivalent (characteristic, for example, of the closely related to Fulgoridae family Dictyopharidae) by its morphology and location at metaphase I. On the other hand, this XY pair appears to be identical to the XB pair described by Kirillova and Kuznetsova (1990) in *Javesella pellucida*, *Criomorphus borealis*, and *Saccharosydne procera*. According to Nokkala et al. (2003: 331), “the evolutionary dynamics of B-chromosomes, that is, the ability to transform into A chromosomes or vice versa, might have played a much more important role in the evolution of karyotypes than previously understood”.

Noteworthy is the different behaviour of B chromosomes in the leafhoppers *Alebra albostriella* and *Alebra wahlbergi* (Kuznetsova et al. 2013). In these species, B-chromosome(s) did not connect to the univalent X chromosome at MI, and when there were two B chromosomes in a set, they did not pair and passed randomly through meiosis as univalents, being still maintained in the populations by unknown means.

### Other cases of polymorphism

Fission and fusion of holokinetic chromosomes do not result in unbalanced meiotic products, and so these rearrangements may be preserved through generations and establish variations in chromosome number within populations. Yet, descriptions of chromosomal polymorphisms are quite rare in Auchenorrhyncha. One can anticipate that it is due to very few studies at the population level in this group. However some chromosomal polymorphisms (other than polymorphism for B-chromosomes) do occur in natural populations of leafhoppers and planthoppers.

**Polymorphism for sex chromosomes.** Some cases of sex chromosome polymorphism were discovered in the leafhoppers *Austragalloides* sp. (Whitten 1968), *Parabolocratus albomaculatus* Distant (Manna and Bhattacharya 1973), *Oncopsis tristis* Zetterstedt, and *Oncopsis flavicollis* (Linnaeus) (John and Claridge 1974), as well as in the planthoppers *Dicranotropis hamata* Boheman (Delphacidae) and *Repetekia orbicularis* Oshanin (Dictyopharidae) (Halkka 1959, Kuznetsova 1986). In *Parabolocratus albomaculatus* from India, the males were found to be dimorphic in sex chromosome constitution. Out of 30 males studied, 16 were of an X(0) type (designated as Type A), while 14 were of an XY type (designated as Type B). Type A was characterized by 17 chromosomes in the spermatogonial complement, with a pair of conspicuously large autosomes and a single medium-sized X chromosome. Type B displayed 2n = 18 with X being the largest, Y relatively small, and the pair of large autosomes present in Type A was absent. The sex chromosomes in Type B were suggested to have originated as neo-X and neo-Y by the X-autosome translocation from Type A (Manna and Bhattacharya 1973).

A very interesting example of sex chromosome polymorphism was revealed by John and Claridge (1974) in British populations of *Oncopsis flavicollis*. In this species, mountain populations occurring on *Betula pubescens* Ehrhart were X(0)-monomorphic, whereas populations in lowland woodlands were polymorphic, containing a mixture of X(0) and neo-XY males in the same or different populations.

Halkka (1959) studied a Finnish population of *Dicranotropis hamata* and found that some males in this population displayed a Y chromosome, while others did not. He suggested that the absence of Y chromosome in these males was a result of its loss, the Y being still inherited in part of the population.

**Polymorphism for autosomes.** Some impressive cases of a fission/fusion polymorphism for autosomes have been described in the Australian leafhopper species *Deltocephalus longuinquus* Kirkaldy (Whitten 1965) and *Alodeltocephalus draba* Evans (Whitten and Taylor 1968), as well as in Greek populations of *Alebra albostriella* and *Alebra wahlbergi* (Kuznetsova et al. 2013). In eight studied populations of *Alodeltocephalus draba*, specimens appeared invariable in having one of the following four chromosome complements: (1) three bivalents + X, (2) four bivalents + X, (3) two bivalents + one trivalent + X, and (4) one bivalent + one tetravalent + X. The reduction in the number of chromosomes has reached different stages in different areas. At Lake Pedder, the chromosome number was almost fixed (2n = 7, three bivalents + X), while at Bruny Island, there occurred a cline in chromosome number decreasing from north to south. This cline was caused by differences in the frequency of chromosome fusions. *Alodeltocephalus draba* was suggested to be under a process of speciation driven by the reorganization of chromosomes that is initiated in some local populations through the fixation of a particular chromosome rearrangement (Whitten and Taylor 1968).

The brown planthopper *Nilaparvata lugens* Stål (Delphacidae) is the only auchenorrhynchan species studied cytogenetically both from natural populations and laboratory cultures. It is notable that natural populations of this species across a wide geographic range revealed almost no instances of chromosomal polymorphism (den Hollander 1982), whereas males from the stock cultures showed a great amount of polymorphism (Liquido 1986, Goh et al. 1992). These differences in the level of chromosomal polymorphism between natural populations and laboratory cultures deserve further investigation.

### Meiosis in males and females

**Meiosis in normal spermatogenesis.** Within Hemiptera, some very interesting and highly aberrant chromosome cycles and anomalous types of meiosis occur in aphids, scale insects, whiteflies, and true bugs, including moss bugs (Coleorrhyncha) (White 1973, Blackman and Hales 1986, Normark 2003, Papeschi and Bressa 2006, Kuznetsova et al. 2011, 2015b). In contrast to all these insects, meiosis in Auchenorrhyncha is essentially simple and uniform in different species and follows the classical “pre-reductional” scheme: during first meiotic division homologous chromosomes undergo pairing, synapsis and recombination at prophase I and segregation at anaphase I. As with autosomes, sex chromosomes undergo pre-reductional meiosis. During second division, sister chromatids separate and migrate to opposite poles at anaphase II creating then haploid daughter cells.

**The number of chiasmata in bivalents.** It is common knowledge that in meiosis, chiasmata (presumed to be the points of genetic crossing-over) are formed uniting homologous chromosomes together until their separation in the reductional division. In most organisms there are one to three chiasmata per bivalent, although in some organisms the number of chiasmata in a bivalent (i.e. the chiasma frequency) varies considerably being typically higher in plants than in animals (White 1973). Halkka (1964) analyzed the number of chiasmata in males and females of species belonging to six families of Auchenorrhyncha, namely, Cicadellidae, Cixiidae, Delphacidae, Issidae, Cercopidae, and Membracidae. In all species, bivalents were found to display one or occasionally two chiasmata, and no great differences in chiasma frequencies were detected between males and females, as well as between leafhoppers and planthoppers.

Similarly, the low number of chiasmata (estimated to be 1-2 from cytogenetic analyses) is a rule in psyllids (Maryańska-Nadachowska 2002) and true bugs (Kuznetsova et al. 2011) and is suggested to represent one of the peculiar features of holokinetic bivalents as such (Nokkala et al. 2004). Based on a detailed analysis of meiosis in a psyllid species, *Beopelma foersteri* Flor, Nokkala et al. (2004) concluded that the cells carrying more than two chiasmata were inevitably eliminated creating thus a strong selection against the formation of multiple chiasmata in holokinetic bivalents. The main cause of this pattern was suggested to be a specific condensation process inherent to holokinetic chromosomes. It is worth noting that bivalents with multiple chiasmata have been observed occasionally in holokinetic groups, including Auchenorrhyncha (see for references Kuznetsova et al. 2009b); however, these observations never advanced beyond metaphase I of spermatogenesis, and therefore, the further fate of the cells with multichiasmate bivalents remained unknown.

**Meiosis in normal oogenesis.** In comparison to the rather abundant data available on male meiosis in Auchenorrhyncha, there have been no comprehensive investigations of chromosome behaviour in female meiosis. The only exceptions are the few descriptions of meiosis in parthenogenetic forms (see section “**Polyploidy**”) and the studies done by Halkka (1959, 1964) on female meiosis of several bisexual species. First, Halkka (1964) provided evidence for the low chiasma frequencies in females. Second, he revealed that females of the leafhopper species *Athysanus argentarius* Metcalf displayed pre-reductional meiosis both for autosomes and sex chromosomes and one chiasma per bivalent (in contrast to two chiasmata in males) (Halkka 1959).

**Meiotic abnormalities.** It is to be noted that the apparent uniformity of meiosis in Auchenorrhyncha could be due to the small number of species which have been studied in any detail. The incidence of meiotic abnormalities and their relationship with different spermatogenic parameters was assessed in the leafhopper species *Alebra albostriella* and *Alebra wahlbergi* (Kuznetsova et al. 2013). Several isolated populations of these species in Greece sampled from different food plants, such as *Castana sativa* Mill., *Fagus sylvatica* L., *Quercus cerris* L., *Acer opalus* Mill., and *Ulmus* spp., showed a great deal of meiotic abnormalities in males, including end-to-end non-homologous chromosomal associations, heterozygous translocation chains, univalents, anaphasic laggards, and aberrant sperms. The primary causes of abnormal chromosome behavior in studied populations, whether those are male-specific meiotic mutations or some environmental mutagens, remained unknown. Also it is not known whether these meiotic abnormalities may play a role in the genome diversity and karyotype evolution of the genus *Alebra*. The resolution of the issues will have to await further studies (Kuznetsova et al. 2013).

### New approaches to cytogenetic studies

White (1978) estimated that over 90% of all speciation events are accompanied by karyotypic changes. Current evidence shows that chromosome numbers in Auchenorrhyncha are quite often remarkably conservative within a group despite the theoretical capacity of holokinetic chromosomes for fusion and fragmentation. It is to be noted, however, that, in general, cytogenetic studies of Auchenorrhyncha use standard techniques, providing evidence for chromosome numbers, sex chromosome mechanisms, and, in outline, the behaviour of chromosomes during meiosis. Nevertheless, for a student of auchenorrhynchan cytogenetics (as well as for an investigator of any other holokinetic group), the main challenge is the identification of individual chromosomes and chromosomal regions. This information would result in considerable progress in the field because it will allow identification of the interchromosomal and, what is more important, intrachromosomal rearrangements involved in the evolution of holokinetic organisms.

Chromosome banding is a staining technique to reveal differentiation within chromosomes as a series of reproducible cross-bands. Besides the identification of individual chromosomes in a karyotype, the bands tell a good deal about fundamental aspects of the chromatin organization and compartmentalization of the genome. These techniques have had an invaluable impact on plant and animal cytogenetics but still are very little used in Auchenorrhyncha. In this group, a number of studies have applied some conventional techniques, such as C-banding, AgNOR-banding, and DNA base specific fluorochrome-banding. C-banding characteristically reveals the extent and location of heterochromatic segments (C-bands), which contain highly condensed, repetitive and largely transcriptionally silent DNA. Fluorochrome-banding mainly involves GC-specific antibiotic chromomycin A_3_ (CMA_3_) and AT- specific 4-6-diamidino-2-phenylindole (DAPI) to detect variation in base composition along the chromosomes. AgNOR-banding reveals the nucleolus organizer regions (NORs), containing the genes that code for ribosomal RNA. These techniques have proved their utility for comparative purposes at the generic level. For example, the C-banding technique showed that taxonomically related species sharing the same chromosome number differ often in chromosome constitution due basically to the accumulation of many rearrangements since divergence from the common ancestor. For instance, differences in C-banding pattern were described between the delphacids *Nilaparvata lugens* and *Calligypona pellucida* Horváth (Noda and Tatewaki 1990), between the cicadas *Tibicen bihamatus* Motschulski and *Platypleura kuroiwae* Matsumura (Perepelov et al. 2002), between the species of the spittlebug genus *Philaenus* (Kuznetsova et al. 2003, Maryańska-Nadachowska et al. 2008, 2012), and between the species of the family Issidae (Kuznetsova et al. 2009b, 2010). The issid species *Hysteropterum albaceticum* Dlabola and *Agalmatium bilobum* Fieber, both with 2n = 26 + X(0) in males, were shown to differ considerably in the amount of C-heterochromatin, which appeared clearly more abundant in the first of these species. The species differed also in C-heterochromatin distribution along the karyotypes and its ability to stain with DAPI and CMA_3_ (Kuznetsova et al. 2009b). On the other hand, the application of AgNOR-banding showed that *Hysteropterum albaceticum* and *Agalmatium bilobum* were similar in having NORs located sub-terminally in the largest pair of autosomes. It should be noted, that such a location of NORs seems to represent the most common pattern in Auchenorrhyncha as a whole (Kuznetsova et al. 2003, 2009b, 2010, Maryańska-Nadachowska et al. 2006, 2012).

In the last few decades, the ability to identify individual chromosomes in a karyotype has been markedly improved by the development of molecular cytogenetic techniques. These include, for example, fluorescence *in situ* hybridization (FISH) to locate the positions of different genes and specific DNA sequences on chromosomes, comparative genomic hybridization (CGH) for analyses of genome homology, genomic *in situ* hybridization (GISH) to identify alien chromosomes or segments, and immunofluorescence to detect the location and relative abundance of the proteins. Some of these techniques have been applied to economically important holokinetic species (Mandrioli et al. 2003, Mandrioli and Borsatti 2007, Marec et al. 2010, Grozeva et al. 2010, 2015), but have not yet been developed specifically for Auchenorrhyncha. The only exceptions are the Southern hybridization of genomic DNA with a telomeric probe and FISH of chromosomes with telomeric and ribosomal probes, which have been applied successfully to several auchenorrhynchan species (Frydrychová et al. 2004, Maryańska-Nadachowska et al. 2013, Golub et al. 2014, Kuznetsova et al. 2015a).

Telomeres are defined as the regions of the chromosomal ends that are required for complete replication, meiotic pairing, and stability of a chromosome (Zakian 2012). The molecular structure of telomeres is characterized by a tandem repeat of a short DNA sequence that is diversely differentiated in eukaryotes. Comparative analysis of these repeats (motifs) in various groups of organisms showed that they are evolutionarily stable, and, having once appeared during evolution, define taxa and phylogenetic branches of high rank (Traut et al. 2007). Frydrychová et al. (2004), using the Southern Hybridization technique, demonstrated the presence of telomeric TTAGG sequences in the genome of *Calligypona pellucida* (Delphacidae). However, this technique is capable to reveal a sequence but not its chromosomal location within a genome. In contrast, *in situ* hybridization (FISH) is a technique that allows precise localization of specific DNA sequences on chromosomes.

Maryańska-Nadachowska et al. (2013) and Golub et al. (2014) pioneered in applying FISH to Auchenorrhyncha. *In situ* hybridization with the telomeric (TTAGG) and 18S rRNA gene probes was used to study eight species of the genus *Philaenus*, Aphrophoridae (Maryańska-Nadachowska et al. 2013) and *Mapuchea chilensis*, Myerslopiidae (Golub et al. 2014). In most eukaryotic genomes, ribosomal DNA (rDNA) consists of tandemly repeated arrays of three genes (18S, 5.8S, and 28S) encoding nuclear rRNA and separated by internal spacers (Hillis and Dixon 1991). These arrays make up the nucleolus organizing regions (NORs) and can be found clustered in one or several regions of the genome. First, the telomeric repeat probe confirmed that the chromosome ends of *Philaneus* spp and *Mapuchea chilensis* are composed of the (TTAGG)*_n_* nucleotide sequence, a common motif of insect telomeres. This motif was reported in the vast majority of evolutionary lineages in Arthropoda and is suggested to represent an ancestral sequence of telomeres in insects (Sahara et al. 1999, Frydrychova et al. 2004, Lukhtanov and Kuznetsova 2010). Second, the 18S rRNA gene probe showed that in *Mapuchea chilensis* 18S rDNA loci are placed on a medium-sized pair autosome and that *Philaneus* species differ from one another in both number and location of major ribosomal gene loci in their karyotypes. Thus, the application of *in situ* hybridization technique to *Philaenus* species showed an extensive reorganization of their genomes: the ribosomal genes changed repeatedly their relative position along the chromosomes indicating that a large number of rearrangements probably occurred during or soon after the species formation.

### Evolutionary relationships revealed from chromosome data

Given that chromosomes represent morphology at small scale, they can be used in phylogenetics in the same way as other morphological characters and can contribute to clarifying the systematics and phylogeny of a particular group.

Chromosome data have contributed to establishing the evolutionary relationships in several different ways which, except for rare occasions (e.g. Blackman 1980, Emeljanov and Kirillova 1990, 1992, Gokhman 2006, Angus and Tatton 2011; for some interesting references see also Lukhtanov and Kuznetsova 2010), are usually ignored by entomologists. As in other insect groups, cytogenetic data have been applied at both higher and lower rank levels in taxonomic and/or phylogenetic studies in Auchenorrhyncha.

**Evolution at and above family level.** Cytogenetic data placed in a phylogenetic context can provide insights into chromosome evolution within a higher rank taxon. A number of successful examples of this approach have been made in Auchenorrhyncha (e.g., Emeljanov and Kirillova 1990, 1992, Kuznetsova et al. 2009a, Maryańska-Nadachowska et al. 2012). One of those deals with the planthopper subfamily Orgeriinae (Dictyopharidae). This group comprises 192 species in 37 genera of four tribes: the Palaearctic ones Ranissini (7 genera, 43 species), Colobocini (1 genus, 1 species), and Almanini (20 genera, 104 species), and the Nearctic tribe Orgeriini, with 37 species in 10 genera (Emeljanov 1980, Emeljanov et al. 2005). For construction and substantiation of the phylogeny of Orgeriinae, three types of characters were used: morphological (Emeljanov 1980), incomplete cytogenetic (Kuznetsova 1986, Kuznetsova et al. 2009a), and preliminary molecular (Emeljanov et al. 2005). As a whole, chromosome complements of 30 species (more than 15% of the total species recognized in Orgeriinae), belonging to 17 genera (almost one-half of the total) and all tribes, except for the African monotypic tribe Colobocini, are currently known. Chromosome numbers being combined with some anatomical data (testis structure in terms of the number of testicular follicles) provided a strong support for the monophyly of Orgeriinae and the recognition of two tribes, Ranissini and Almanini. All Ranissini were shown to have 26 autosomes, a simple sex chromosome mechanism of an X(0) type (2n = 26 + X), and male testes each composed of 6 follicles. On the other hand, species belonging to Almanini were described as having a pair of autosomes less, a secondary neo-XY sex determining system (2n = 24 + neo-XY), and 4 follicles per male testis. The karyotype of Ranissini was suggested to have evolved by the fusion of two autosome pairs in an ancestral karyotype of 2n = 28 + X(0) (inherent to the second subfamily Dictyopharinae). The karyotype of Almanini, in its turn, had originated from that of Ranissini by an X-autosome fusion. Besides the gradual reduction of the total number of autosome pairs, there is apparently a trend towards the reduction of the number of testicular follicles in the evolution of Dictyopharidae. Thus, karyotype and testis structure both suggest a “basal” position of Ranissini within Orgeriinae. The states of these characters encountered in Almanini (2n = 24 + neo-XY and 4 follicles) are treated as being derived from those of Ranissini (2n = 26 + X and 6 follicles), which is in good agreement with the morphological data (Emeljanov 1980). The point of interest is the tribe Orgeriini. The latter shows a number of morphological apomorphies and, therefore, is considered to be one of the most advanced tribes within Orgeriinae (Emeljanov 1980, Emeljanov et al. 2005). Despite of this, three recently studied species of Orgeriini (*Orgerius rhyparus* Stål, *Orgerius ventosus* Ball & Hartzell, and *Deserta bipunctata* Ball) were found to share the same karyotype and testis structure as the basal tribe Ranissini (Kuznetsova et al. 2009a).

Additional examples showing the significance of chromosome data for the systematics and phylogenetics of Auchenorrhyncha are given below. As noted above, Fulgoromorpha differ distinctly from Cicadomorpha in chromosome numbers (Fig. 8). The planthopper families Fulgoridae and Dictyopharidae are also a good case in point. Emeljanov (1979) identified a number of important morphological differences that support the discreteness of these families. Fulgoridae and Dictyopharidae are also distinguished by the morphology and the assumed origin of their Y chromosomes (Kuznetsova et al. 2009a; see also sections “**Sex determining systems**” and “**Polymorphism for B-chromosomes**”). Opinions vary broadly on the phylogenetic position of the planthopper family Tettigometridae. Although this family is mainly accepted as the most basal one within Fulgoroidea, some morphological and molecular evidence suggest that it is a relatively derived lineage among fulgoromorphans (see for references Yeh et al. 2005 and Urban and Cryan 2007). The currently available chromosome data seem to be consistent with this opinion: all hitherto studied species of Tettigometridae display the evolutionarily derived neo-XY sex chromosome system (Kuznetsova and Kirillova 1990, Kirillova 1993).

**Evolution below family level.** Cytogenetic data also provide useful information about lower rank taxonomic relationships. For example, in the leafhopper *Rhopalopyx preyssleri* Herrich-Schäffer, Halkka (1959) discovered two types of populations in Finland, i.e. with 2n = 14 + X and with 12 + XY in males respectively. He treated these differences in terms of a common polymorphism for an X-autosome translocation resulting in the formation of a neo-XY system. However, shortly afterwards, Vilbaste (1962) showed that the putative “12 + XY race” of *Rhopalopyx preyssleri* was in fact *Rhopalopyx adumbrata* Sahlberg.

Whitten (1965) described variation in chromosome number in different populations of a leafhopper species in Australia. An examination of male genitalia by J.W. Evans revealed that two morphologically distinct species were present. The 2n=11 group belonged to a species which, subsequent to Whitten’s (1965) paper, was separated from the genus *Deltocephalus* as *Alodeltocephalus longuinquus* Kirkaldy, while the remaining chromosomal groups were morphologically uniform and all included in the new species, *Alodeltocephalus draba* (Evans 1966).

The meadow spittlebug genus *Philaenus* (Aphrophoridae) is likewise a good example. This genus has been studied using morphological (Drosopoulos and Remane 2000, Drosopoulos 2003), molecular (Maryańska-Nadachowska et al. 2010, Seabra et al. 2010) and cytogenetic (Kuznetsova et al. 2003, 2015a, Maryańska-Nadachowska et al. 2008, 2012, 2013) techniques. Numerous studies have explored the outstanding colour polymorphism and systematics of this genus (e.g. Drosopoulos and Remane 2000, Drosopoulos 2003). A total of eight *Philaenus* species are presently recognized, including the Mediterranean species *Philaenus tesselatus* Melichar, *Philaenus loukasi*, *Philaenus arslani* Abdul-Nur & Lahoud, *Philaenus signatus* Melichar, *Philaenus maghresignus* Drosopoulos & Remane, *Philaenus tarifa* Remane & Drosopoulos, and *Philaenus italosignus* Drosopoulos & Remane, and the Holarctic species *Philaenus spumarius* Linnaeus (Drosopoulos and Remane 2000). Based on morphology, the genus is currently divided into two groups: the *“spumarius”* species group and the *“signatus”* species group (Drosopoulos and Remane 2000), whereas based on larval food plant preferences, the genus is divided into three ecological groups: developing on the lily *Asphodelus aestivus* Brot., on xerophilic plants, and on various dicotyledonous and monocotyledonous plants (Drosopoulos 2003).

The results of a recent phylogenetic study of *Philaenus* using nucleotide sequences from two mitochondrial (*COI* and *CytB*) genes and one nuclear (*ITS2*) region are in general agreement both with the morphological and the food plant preferences groupings (Maryańska-Nadachowska et al. 2010). Likewise, differences in karyotype were found to be largely in agreement with the recognized groupings proposed on the basis of morphology and on the basis of larval food plant relationships. Cytogenetic analysis has revealed that a number of *Philaenus* species share the same karyotype while some others differ in chromosome number, sex chromosome system and additional cytogenetic characters. The species feeding on *Asphodelus aestivus* were shown to have 2n = 22 + neo-XY (*Philaenus signatus*, *Philaenus maghresignus* and *Philaenus tarifa*) or 2n = 20 + neo-neo-X_1_X_2_Y (*Philaenus italosignus*). These species are included into the species group *“signatus”*. Among the species of the *“spumarius”* group, *Philaenus loukasi* and *Philaenus arslani*, with larvae developing on arid plants, share 2n = 18 + neo-XY, whereas *Philaenus tesselatus* and the polyphagous species *Philaenus spumarius*, feeding on a wide range of dicotyledonous plants, possess 2n = 22 + X(0). It has been postulated that the ancestral karyotype of *Philaenus* is 2n = 24 + X(0) and that karyotype changes occurred several times independently in the genus (Maryańska-Nadachowska et al. 2012, 2013).

In conclusion, it may be said that one of the most important ways of increasing the taxonomic and phylogenetic inferences based on chromosome data is to enlarge sampling of taxa. Considerable progress in our understanding of the cytogenetics of Auchenorrhyncha will come from the development and application of new molecular cytogenetic techniques, which appear clearly advantageous for revealing important markers in holokinetic chromosomes. These techniques are expected to provide useful insights into the genome constitution and mechanisms of karyotype evolution in this large group of Hemiptera.
